# Endovascular Thrombectomy with or without Intravenous Thrombolysis for Anterior Circulation Large Vessel Occlusion in the Imperial College London Thrombectomy Registry

**DOI:** 10.3390/jcm12031150

**Published:** 2023-02-01

**Authors:** Lucio D’Anna, Matteo Foschi, Michele Russo, Tsering Dolkar, Orsolya Vittay, Luke Dixon, Paul Bentley, Zoe Brown, Charles Hall, Omid Halse, Sohaa Jamil, Harri Jenkins, Dheeraj Kalladka, Joseph Kwan, Abid Malik, Maneesh Patel, Neil Rane, Dylan Roi, Abhinav Singh, Marius Venter, Kyriakos Lobotesis, Soma Banerjee

**Affiliations:** 1 Department of Stroke and Neuroscience, Charing Cross Hospital, Imperial College London NHS Healthcare Trust, London W6 8RF, UK; 2 Department of Brain Sciences, Imperial College London, London SW7 2AZ, UK; 3 Department of Biotechnological and Applied Clinical Sciences, University of L’Aquila, 67100 L’Aquila, Italy; 4 Department of Cardiology, S.Maria dei Battuti Hospital, AULSS 2 Veneto, 31015 Conegliano, Italy; 5 Neuroradiology, Department of Imaging, Charing Cross Hospital, Imperial College London, NHS Healthcare Trust, London W6 8RF, UK

**Keywords:** mechanical thrombectomy, intravenous thrombolysis, large vessel occlusion, ischemic stroke

## Abstract

**Background and purpose.** Mechanical thrombectomy (MT) is the standard of care for eligible patients with a large vessel occlusion (LVO) acute ischemic stroke. Among patients undergoing MT there has been uncertainty regarding the role of intravenous thrombolysis (IVT) and previous trials have yielded conflicting results regarding clinical outcomes. We aim to investigate clinical, reperfusion outcomes and safety of MT with or without IVT for ischemic stroke due to anterior circulation LVO. **Materials and Methods.** This observational, prospective, single-centre study included consecutive patients with acute LVO ischemic stroke of the anterior circulation. The primary outcomes were the rate of in-hospital mortality, symptomatic intracranial haemorrhage and functional independence (mRS 0–2 at 90 days). **Results.** We enrolled a total of 577 consecutive patients: 161 (27.9%) were treated with MT alone while 416 (72.1%) underwent IVT and MT. Patients with MT who were treated with IVT had lower rates of in-hospital mortality (*p* = 0.037), higher TICI reperfusion grades (*p* = 0.007), similar rates of symptomatic intracranial haemorrhage (*p* = 0.317) and a higher percentage of functional independence mRS (0–2) at 90 days (*p* = 0.022). Bridging IVT with MT compared to MT alone was independently associated with a favorable post-intervention TICI score (>2b) (OR, 1.716; 95% CI, 1.076–2.735, *p* = 0.023). **Conclusions.** Our findings suggest that combined treatment with MT and IVT is safe and results in increased reperfusion rates as compared to MT alone.

## 1. Introduction

Mechanical thrombectomy (MT) is the standard of care for eligible patients with a large vessel occlusion (LVO) acute ischemic stroke [1]. Among patients undergoing MT there has been uncertainty regarding the role of the intravenous thrombolysis (IVT). Although thrombolytic agents, such as alteplase, may contribute to early reperfusion, IVT could delay the MT, increase the risk of symptomatic intracerebral hemorrhage, or distal migration of thrombi, rendering them inaccessible to MT. Previous trials have addressed this question but have yielded conflicting findings regarding clinical outcomes [2,3,4,5]. The SKIP trial in Japan and the MR CLEAN-NO IV trial in Europe did not show non-inferiority of endovascular therapy alone [2,3], whereas the DEVT and DIRECT-MT trials, both in China, showed non-inferiority, but used wide margins inclusive of clinically meaningful effects [4,5]. Indeed, the DEVT allowed for up to a 10% reduced rate of functional independence, and DIRECT-MT allowed for an adjusted common odds ratio of as low as 0·80 for a favorable functional level, for direct endovascular therapy to be declared non-inferior. Both trials also had methodological issues that increased the risk of bias, including long arrival to intravenous thrombolysis start times and significant protocol deviations for DIRECT- MT. Nevertheless, the results generated the hypothesis of differential treatment effects in Chinese patients, or perhaps Asian patients, compared with others. Two further trials by Fischer and colleagues (SWIFT-DIRECT) [6] and Mitchell and colleagues (DIRECT-SAFE) [7] have further tested the non-inferiority of bypassing intravenous thrombolysis. These two trials did not show the non-inferiority of omitting intravenous thrombolysis. Moreover, in both trials, the directions of effects favored intravenous thrombolysis use.

In our study, we aim to investigate the clinical, reperfusion, and safety outcomes of MT with and without IVT in patients with acute LVO ischemic stroke of the anterior circulation in a real-world setting.

## 2. Methods

In this observational, investigator-initiated, prospective study, we recruited stroke patients consecutively treated with MT at the Stroke Department, Charing Cross Hospital, Imperial College Healthcare NHS Trust, London between 1 January 2016 and 30 June 2021. This study has obtained approval from the UK Health Regulator Authority (HRA) (HRA Reference No.: 275260). The study has also received confirmation of capacity and capability from the Imperial College Healthcare NHS Trust. The study was conducted in accordance with the recommendations for physicians involved in research on human subjects adopted by the 18th World Medical Assembly, Helsinki 1964 and later revisions.

### 2.1. Organization of the Imperial Stroke Thrombectomy Stroke Network

**Figure 1:**
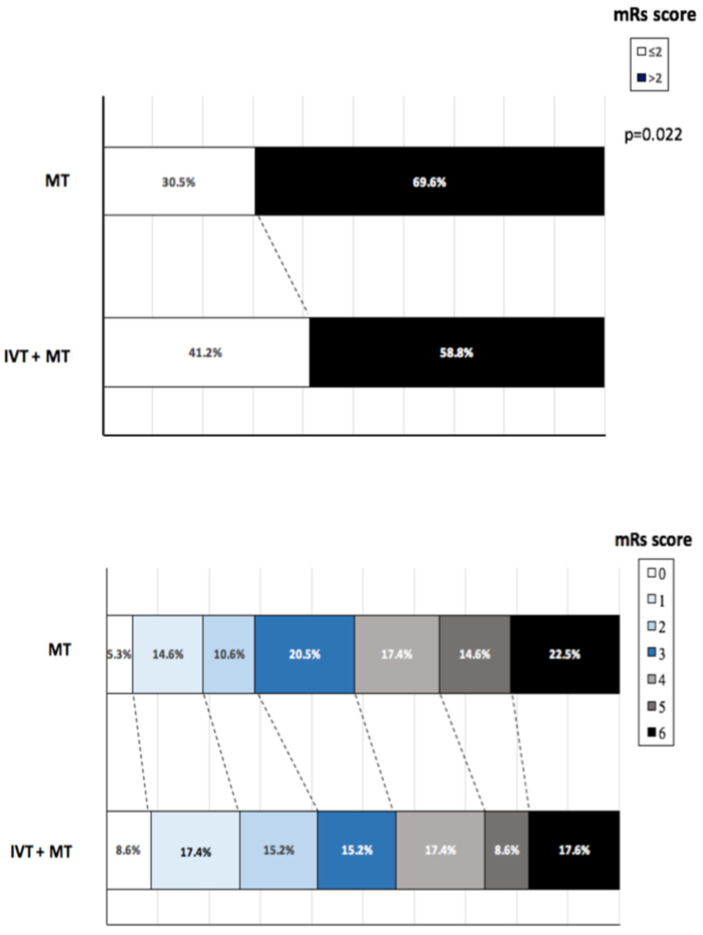
Distribution of modified Rankin Scale (mRS) scores at 90 days.

The Stroke Department at Charing Cross Hospital is the Northwest London (UK) regional Comprehensive Stroke Center (CSC) for MT in an urban metropolitan area with more than 6.4 million people. It cares for over 1800 patients admitted to the HASU annually. Our CSC accepts potential candidates 24/7 for mechanical thrombectomy from the primary stroke centers (PSCs) of our stroke networks (Figure 1). Patients from both PSCs and CSC presenting with features of acute stroke were evaluated in the hyperacute setting with appropriate neuroimaging and vascular imaging when indicated: computed tomography (CT) and computed tomography angiography (CTA). Patients who fulfilled the relevant indications and who were without exclusion criteria would undergo acute recanalization therapy.

### 2.2. Patients Inclusion and Exclusion Criteria for the Analysis

For the purpose of this analysis, the criteria for patients selection were: (1) age ≥ 18 years; (2) NIHSS score 6 or more; (3) Alberta Stroke Program Early CT score (ASPECTS) [8] 5 or more; (4) LVO sites: distal internal carotid artery, middle cerebral artery segments M1 or M2; (5) initiation of the MT had to be possible within 6 h after the stroke onset. IVT was administered in all patients who presented within 4.5 h of stroke symptom onset without contraindications according to the guidelines.

For this analysis, we excluded stroke patients with basilar artery occlusion and patients that met DAWN or DEFUSE 3 eligibility criteria [1,9,10].

### 2.3. Clinical and Radiological Assessments

Baseline demographic and clinical characteristics and pre-admission treatments were collected from patients’ medical records. NIHSS was performed in all patients on admission and 24 h after the MT. The modified Rankin Scale (mRS) was used to assess the patients’ initial pre-stroke statuses, and the level of functional independence at 90 days of the patients was evaluated centrally through a telemedicine consultation or in-person consultation at the CSC or local PSC. Procedural metrics were collected prospectively. The extent of the initial core infarct was determined on pre-therapeutic CT using ASPECTS [8]. In addition, an independent rater (consultant neuroradiologist) who did not participate in the endovascular stroke treatment of included patients evaluated pre-therapeutic CT and follow-up CT at 24 h. Revascularization was assessed by applying the modified thrombolysis in cerebral infarction (TICI) classification [11]. Successful recanalization was defined as grade 2b, 2c, or 3 of reperfusion. Hemorrhagic transformation (HT) was defined on follow-up CT at 24 h according to the Heidelberg Bleeding Classification [12]. In the case of more than one hemorrhagic lesion on brain scan, the worst possible category was assumed. HT was considered symptomatic if it was not seen on the admission brain scan and there was, subsequently, a suspicion of hemorrhage or a decline in neurological status (an increase of more than 4 points in the NIHSS). Functional independence was defined as mRS scores of 0 through 2.

Clinical outcomes (in-hospital mortality, malignant middle cerebral artery stroke, NIHSS at 24 h) were also prospectively documented.

### 2.4. Statistical Analysis

Categorical variables were summarized and evaluated in percentages. Continuous variables were summarized as mean ± standard deviation (SD). Shapiro–Wilk and Kolmogorov–Smirnov tests were used to test the normality of variables, while variance homogeneity was tested using Levene’s test. Continuous variables were compared between groups using Student’s t-test and the Mann–Whitney test, while nominal or categorical variables were compared between groups using Fisher’s exact test and Pearson χ^2^ test. Univariate regression analysis was performed including all demographic and clinical characteristics. A multivariate logistic regression analysis including variables with statistical significance < 0.05 was performed to identify independent predictors of in-hospital death, favorable post-intervention TICI score, and 90-day functional independence. Results were reported as Odds Ratio (OR) with 95% confidence intervals. The significance level was set at *p* < 0.05. Statistical analysis was performed using SPSS statistical package, version 20.0 (SPSS Inc. Chicago; III., USA).

## 3. Results

A total of 577 patients who underwent MT within 6 h from the time they were last known to be well (Table 1) were included in this analysis. A total of 416 patients (72.09%) also underwent IVT. The two groups did not significantly differ respect to baseline NIHSS score and ASPECTS score on admission. Patients who received IVT with MT treatment had a lower rate of atrial fibrillation or flutter (*p* < 0.001), congestive heart failure (*p* = 0.035), peripheral artery disease (*p* < 0.001), and prior stroke (*p* < 0.001), but a higher rate of symptomatic carotid artery disease (*p* = 0.034). Between the two groups, there was not a statistically significant difference in terms of distribution of the pre-stroke mRS (*p* = 0.090). As expected, a lower proportion of patients who received both MT and IVT were taking anticoagulants compared to those who received MT only (*p* =<0.001). The time gap between the admission at our CSC and groin puncture was significantly shorter in MT patients treated with IVT compared to patients without IVT (54 min versus 79 min, *p* < 0.001). The NIHSS score on admission, the ASPECT score, thrombus locations, and type of anesthesia used were similar. On discharge, patients treated with only MT were taking more anticoagulant compared to those treated with IVT and MT (*p* =<0.001).

Outcomes are reported in Table 2. Patients with MT who were treated with IVT had lower rates of in-hospital mortality (*p* = 0.037) and had higher TICI reperfusion grades (*p* = 0.007). Patients treated with IVT and MT showed similar rates of malignant MCA infarct, HT at 24 h, symptomatic intracranial hemorrhage, and NIHSS at 24 h compared to patients treated only with MT. The distribution of the 90-day mRS is shown in Figure 1. A significantly higher percentage of patients treated with MT and IVT achieved a functional independence at 90 days compared to patients treated with only MT (*p* = 0.022).

Combined treatment with MT and IVT was associated with higher likelihood of successful reperfusion, based on TICI grade 2b or greater (OR, 1.716; 95% CI, 1.076–2.735, *p*= 0.023) (Table 3), while it was not significantly associated with reduced in-hospital mortality (Supplemental Table S1) and functional independence (Supplemental Table S2). Of note, a favorable post-intervention TICI score (>2b) was significantly associated with functional independence at 90-day (OR, 5.383; 95% CI, 2.184–13.275, *p* < 0.001) (Supplemental Table S2).

## 4. Discussion

One of the major findings of our analysis is that bridging IVT with MT was associated with a higher rate of favorable reperfusion outcome (TICI score > 2b) as compared to MT alone, without increasing the risk of intracranial bleeding. Previous clinical trials have questioned the need for intravenous thrombolysis if patients are eligible to undergo MT and the procedure can be performed in a timely fashion. The SKIP trial in Japan [2], MR CLEAN-NO IV trial in Europe [3], and DEVT [4] and DIRECT-MT [13] trials, both in China, and the recently published DIRECT-SAFE trial [7] showed no significant differences between groups in terms of symptomatic intracerebral hemorrhage and rates of successful reperfusion after MT. Yet, our findings are more in line with observations from the recent clinical trial by Fischer and colleagues (SWIFT-DIRECT) [6] which, beyond demonstrating a similar rate of symptomatic intracranial hemorrhage between patients undergoing MT alone and patients receiving IVT plus MT, documented that a successful reperfusion was less common in patients assigned to thrombectomy alone.

Another key finding of our study is that we observed significant differences in terms of in-hospital mortality rate and percentage of patients who achieved functional independence (mRS 0–2) at 90 days in favor of the group of patients treated with bridging therapy compared to MT alone. In our cohort, however, on the respective multivariate logistic regression analysis, combined treatment with MT and IVT did not independently predict the two favorable outcomes. Previous randomized trials [2,3,4,5] and meta-analysis [13] have provided conflicting results on the benefit of direct MT in comparison to the combination of IVT and MT. Recently, the SWIFT-DIRECT [6] trial tested the non-inferiority of bypassing intravenous thrombolysis. The authors concluded that the non-inferiority of thrombectomy alone when compared with intravenous alteplase plus thrombectomy in patients presenting with acute ischemic stroke due to large vessel occlusion could not be shown. IVT can add benefits to highly effective endovascular therapy as it allows immediate administration of a potentially beneficial reperfusion therapy before endovascular therapy eligibility is established and while preparation for the procedure is under way. Moreover, IVT might be the only reperfusion therapy received if intracranial thrombi are inaccessible due to vessel tortuosity or resistant to thrombectomy attempts. Finally, as also demonstrated in our analysis, IVT might improve the rate of technically successful thrombectomy [14]. Therefore, on the basis of the recent evidence, it is recommended to administer intravenous alteplase before thrombectomy in eligible patients [1].

Our work provides one of the largest available single-center real-life data on acute stroke patients with LVO undergoing reperfusion therapies, and contributes to the growing body of evidence on the utility of IVT as a bridging therapy addressing applicability to a UK population. However, our study has several limitations. The non-randomized design is likely to have introduced biases. The reported associations in our non- randomized study could be influenced by numerous potential confounders, even if statistical models were used to adjust for them. Despite our efforts, we cannot exclude that our results could have been influenced by an incomplete adjustment for patient characteristics in selecting the model for the multivariable analysis. Moreover, non-randomized design might have introduced an imbalance of the two sample groups. Information on functional outcome assessment at 90 days could be reached in 524 patients (90.8%). Finally, the study was conducted within a single stroke network.

## 5. Conclusions

Our findings suggest that combined treatment with MT and IVT is safe and results in increased reperfusion rates.

## Figures and Tables

**Table 1:** Characteristics of patients treated with MT with and without IVT.

	Overall (*n* = 577)	MT without IVT (*n* = 161)	MT with IVT (*n* = 416)	*p* Value
Baseline demographics				
Age, years [mean ± standard deviation]	67.4 ± 14.6	69.0 ± 14.7	66.8 ± 14.5	0.098
Male sex [*n*, (%)]	313 (54.2)	79 (49.1)	234 (56.3)	0.120
Cardiovascular risk factors				
Hypertension [*n*, (%)]	324 (56.2)	97 (60.2)	227 (54.6)	0.217
History of uncontrolled hypertension [*n*, (%)]	14 (2.4)	5 (3.1)	9 (2.2)	0.549
Diabetes [*n*, (%)]	113 (19.6)	35 (21.7)	78 (18.8)	0.417
Hypercholesterolemia [*n*, (%)]	264 (45.8)	77 (47.8)	187 (45.0)	0.534
Smoking status [*n*, (%)]				0.170
Never smoker	459 (79.5)	132 (82.0)	327 (78.6)	
Current Smoking	84 (14.6)	17 (10.6)	67 (16.1)	
Former Smoking	34 (5.9)	12 (7.5)	22 (5.3)	
Medical history				
Atrial fibrillation/atrial flutter [*n*, (%)]	249 (43.2)	95 (59.0)	153 (37.0)	<0.001
Coronary artery disease [*n*, (%)]	97 (16.8)	30 (18.6)	67 (16.1)	0.466
Congestive heart failure [*n*, (%)]	52 (9.0)	21 (13.0)	31 (7.5)	0.035
Symptomatic carotid artery disease [*n*, (%)]	61 (10.6)	10 (6.2)	51 (12.3)	0.034
Peripheral artery disease [*n*, (%)]	25 (4.3)	15 (9.3)	10 (2.4)	<0.001
Previous ischemic cerebrovascular events [*n*, (%)]	101 (17.5)	43 (26.7)	58 (13.9)	<0.001
Previous history of intracranial hemorrhage [*n*, (%)]	5 (0.9)	3 (1.9)	2 (0.5)	0.108
Dementia [*n*, (%)]	4 (0.7)	0 (0.0)	4 (1.0)	0.580
Pre-stroke mRS [median (IQR)]				0.090
		0 (0–0)	0 (0–0)	
Preadmission medications				
Anticoagulant [*n*, (%)]	96 (16.7)	74 (46.0)	22 (5.3)	<0.001
Antiplatelet [*n*, (%)]	134 (23.2)	31 (19.3)	103 (24.8)	0.286
Aspirin	86 (15.2)	22 (13.8)	64 (15.8)	
Clopidogrel	38 (6.7)	6 (3.8)	32 (7.9)	
DAPT	10 (1.8)	3 (1.9)	7 (1.7)	
Statin [*n*, (%)]	195 (34.8)	60 (38.2)	135 (33.4)	0.284
Antihypertensive [*n*, (%)]	292 (51.7)	92 (57.9)	200 (49.3)	0.066
Drip and Ship, *n*, (%)	335 (58.1%)	255 (61.3%)	80 (49.6%)	0.011
Time intervals				
Onset to needle (minutes) [mean ± standard deviation]	-	-	130.6 ± 54.7	-
Door to needle time (minutes) [mean ± standard deviation]	-	-	42.4 ± 29.7	-
Onset to groin puncture time (minutes) [mean ± standard deviation]	-	305.4 ± 162.7	276.1 ± 80.7	0.069
Door to groin puncture time (minutes) [median (IQR)]	-	79.0 (43.0–117.0)	54.0 (39.0–98.0)	0.001
Stroke characteristics				
NIHSS on admission [median (IQR)]	18.0 (13.0–21.0)	17.0 (13.0–21.0)	18.0 (13.0–21.0)	0.309
ASPECTS score [median (IQR)]	8.0 (7.0–9.0)	8.0 (7.0–9.0)	8.0 (7.0–9.0)	0.635
Thrombus location [*n*, (%)]				0.327
ICA	32 (5.5)	10 (6.2)	22 (5.3)	
M1	310 (53.7)	85 (52.8)	225 (54.1)	
M2	84 (14.6)	27 (16.8)	57 (13.7)	
ICA + M1	115 (19.9)	34 (21.1)	81 (19.5)	
M1 + M2	36 (6.2)	5 (3.1)	31 (7.5)	
Anesthesia used, *n* (%)				0.441
GA		95 (59)	243 (58.6)	
LA		65 (40.4)	163 (39.3)	
Converted LA to GA		1 (0.6)	9 (2.2)	
Hyper acute treatment				
Thrombolytic agent, *n* (%)				
*Alteplase*		–	403 (96.9)	–
*Tenecteplase*		–	13 (3.1)	–
Thrombo-aspiration [*n*, (%)]		105 (65.2)	262 (63.0)	0.690
Stent retriever [*n*, (%)]		84 (52.2)	193 (46.4)	0.222
Combination of aspiration/stent retriever [*n*, (%)]		49 (30.4)	107 (25.7)	0.241
Proximal balloon flow [*n*, (%)]		15 (9.3)	40 (9.6)	0.873
Distal catheter [*n*, (%)]		129 (80.1)	336 (80.8)	0.574
Discharge medications				
Anticoagulant [*n*, (%)]	218 (38.0)	81 (50.3)	137 (33.2)	<0.001
Antiplatelet [*n*, (%)]	390 (67.6)	112 (69.6)	278 (66.8)	0.851
Aspirin	354 (62.4)	101 (63.9)	253 (61.9)	
Clopidogrel	12 (2.9)	3 (1.9)	9 (2.2)	
DAPT	24 (4.2)	8 (5.1)	16 (3.9)	
Statin [*n*, (%)]	320 (57.3)	94 (59.9)	226 (56.4)	0.450
Antihypertensive [*n*, (%)]	257 (45.8)	90 (57.3)	167 (41.3)	0.001

Legend: MT: mechanical thrombectomy; IVT: intravenous thrombolysis; mRS = modified Rankin Scale; DAPT = dual antiplatelet therapy; NIHSS = National Institutes of Health Stroke Scale; ASPECTS = The Alberta Stroke Program Early CT Score; DAPT = dual antiplatelet therapy; ICA = internal carotid artery; IQR = interquartile range; GA; general anesthesia; LA: local anesthesia.

**Table 2:** Outcomes in patients treated with MT with and without IVT.

	Overall (*n* = 577)	MT without IVT (*n* = 161)	MT with IVT (*n* = 416)	*p* Value
In-hospital mortality [*n*, (%)]	49 (8.4)	20 (12.4)	29 (7.0)	0.037
Malignant MCA infarct [*n*, (%)]	61 (10.6)	20 (12.4)	41 (9.9)	0.378
NIHSS at 24 h [median (IQR)]	11 (5–20)	13 (6–22)	11 (5–19)	0.180
HT at 24 h [*n*, (%)]				0.600
1a	30 (5.2)	8 (5.0)	22 (5.2)	
1b	46 (8.0)	16 (9.9)	30 (7.2)	
1c	9 (1.6)	3 (1.9)	6 (1.4)	
2	13 (2.3)	2 (1.2)	11 (2.6)	
3a	3 (0.5)	0 (0.0)	3 (7.2)	
3b	3 (0.5)	0 (0.0)	3 (7.2)	
3c	17 (2.9)	5 (3.1)	12 (2.9)	
3d	0 (0.0)	0 (0.0)	0 (0.0)	
S-ICH [*n*, (%)]	22 (3.8)	4 (2.5)	18 (4.3)	0.317
TICI grade				0.007
(0, 1, 2a)		41 (25.4)	66 (15.9)	
(2b, 2c, 3)		120 (74.6)	352 (84.1)	

Legend: MT: mechanical thrombectomy; IVT: intravenous thrombolysis; MCA: middle cerebral artery; HT: hemorrhagic transformation; TICI: modified thrombolysis in cerebral infarction classification; S-ICH: symptomatic intracerebral hemorrhage.

**Table 3:** Univariate and multivariate logistic regression analysis for prediction of a favorable post-intervention TICI score (2b, 2c and 3).

	Univariate Analysis		Multivariate Analysis	
	Or (95% Ci)	*p*-Value	Or (95% Ci)	*p*-Value
IVT plus MT (as compared to MT only)	1.847 (1.181; 2.888)	0.001	1.716 (1.076; 2.735)	0.023
Age (per year)	0.984 (0.969; 0.999)	0.039	0.986 (0.970; 1.002)	0.093
Previous history of intracranial hemorrhage	0.143 (0.024; 0.866)	0.034	0.197 (0.029; 1.319)	0.094
Site of occlusion (ICA as reference)	–	0.020	–	0.039
M1	3.756 (1.730; 8.151)	0.001	3.452 (1.533; 7.774)	0.003
M2	3.456 (1.382; 8.646)	0.008	3.504 (1.352; 9.079)	0.010
ICA + M1	3.611 (1.518; 8.593)	0.004	3.662 (1.489; 9.003)	0.005
M1 + M2	2.992 (1.005; 8.908)	0.049	2.484 (0.804; 7.676)	0.114
Stent retriever	0.448 (0.288; 0.698)	<0.001	0.465 (0.293; 0.737)	0.001

Legend: MT: mechanical thrombectomy; IVT: intravenous thrombolysis.

## Data Availability

Data available upon reasonable request.

## Supplementary Materials

The following supporting information can be downloaded at: https://www.mdpi.com/article/10.3390/jcm12031150/s1, Table S1: Univariate and multivariate logistic regression analysis for prediction of in-hospital death; Table S2: Univariate and multivariate logistic regression analysis for prediction of functional independence at 90 days (mRS 0, 1 and 2).
