# Supplementary material for: Endovascular Thrombectomy with or without Intravenous Thrombolysis for Anterior Circulation Large Vessel Occlusion in the Imperial College London Thrombectomy Registry
Source: J Clin Med. 2023 Feb 1;12(3):1150. doi: 10.3390/jcm12031150 (PMC9918289; doi:10.3390/jcm12031150)
Supplement: Supplementary file 1 [file jcm-12-01150-s001.zip › jcm-2193169-supplementary.pdf]

Supplementary Materials

Supplemental Table S1. Univariate and multivariate logistic regression analysis for prediction of in-hospital death

	Univariate analysis		Multivariate analysis	
	**OR (95% CI)**	***p-value***	**OR (95% CI)**	***p-value***
IVT plus MT (as compared to MT only)	0.531 (0.291; 0.969)	0.039	0.725 (0.332; 1.581)	0.419
History of uncontrolled hypertension (per year)	9.035 (2.998; 27.231)	<0.001	7.797 (1.638; 36.122)	0.009
Diabetes	1.927 (1.010; 3.676)	0.047	2.303 (1.072; 4.949)	0.033
Anticoagulation on admission	2.161 (1.113; 4.194)	0.023	1.523 (0.631; 3.679)	0.350
Statin on admission	1.829 (1.009; 3.318)	0.047	1.242 (0.600; 2.572)	0.559
ASPECT score	0.729 (0.606; 0.877)	0.001	0.735 (0.602; 0.897)	0.003
Stent retriever	1.968 (1.074; 3.606)	0.028	0.780 (0.288; 2.109)	0.624
Combination of aspiration/stent retriever	2.308 (1.251; 4.260)	0.007	2.943 (1.104; 7.849)	0.031
Post-intervention favorable TICI (2b, 2c, 3)	0.376 (0.195; 0.691)	0.002	0.396 (0.187; 0.839)	0.016

Legend: MT: mechanical thrombectomy; IVT: intravenous thrombolysis; TICI:modified thrombolysis in cerebral infarction classification; ASPECTS = The Alberta Stroke Program Early CT Score.

Supplemental Table S2. Univariate and multivariate logistic regression analysis for prediction of functional independence at 90 days (mRS 0, 1 and 2)

	Univariate analysis		Multivariate analysis	
	**OR (95% CI)**	***p-value***	**OR (95% CI)**	***p-value***
IVT plus MT (as compared to MT only)	1.598 (1.068; 2.391)	0.023	1.552 (0.829; 2.907)	0.169
Age (per increasing year)	0.966 (0.954; 0.978)	<0.001	0.970 (0.951; 0.989)	0.002
Hypertension	0.478 (0.334; 0.684)	<0.001	0.735 (0.407; 1.329)	0.309
Prior to stroke mRS (per increasing	0.388 (0.278; 0.542)	<0.001	0.434 (0.270; 0.697)	0.001
Statin on admission	0.611 (0.416; 0.896)	0.012	0.922 (0.491; 1.731)	0.800
Anticoagulation on discharge	1.460 (1.019; 2.092)	0.039	1.707 (1.007; 2.896)	0.047
Statin on discharge	1.524 (1.059; 2.194)	0.023	1.242 (0.600; 2.572)	0.559
NIHSS on admission (per	0.915 (0.885; 0.945)	<0.001	0.902 (0.859; 0.947)	<0.001
ASPECT score	1.309 (1.145; 1.498)	<0.001	1.225 (1.021; 1.470)	0.029
Site of occlusion (reference ICA)	-	0.021		0.146
M1	2.286 (0.953; 5.484)	0.064	3.113 (0.700; 13.843)	0.136
M2	2.927 (1.126; 7.607)	0.028	5.735 (1.181; 27.863)	0.030
ICA + M1	1.353 (0.528; 3.472)	0.529	2.828 (0.576; 13.874)	0.200
M1 + M2	3.429 (1.152; 10.202)	0.027	5.552 (0.946; 32.594)	0.058
Onset to groin (per increasing minute)	0.998 (0.996; 1.000)	0.039	0.998 (0.996; 1.001)	0.201
Stent retriever	0.489 (0.341; 0.702)	<0.001	0.572 (0.296; 1.104)	0.096

Combination of aspiration/stent retriever	0.535 (0.351; 0.815)	0.004	0.980 (0.462; 2.078)	0.957
Post-intervention favorable TICI (2b, 2c, 3)	5.504 (2.848; 10.637)	<0.001	5.385 (2.184; 13.275)	<0.001

Legend: MT: mechanical thrombectomy; IVT: intravenous thrombolysis; ASPECTS = The Alberta Stroke Program Early CT Score; TICI:modified thrombolysis in cerebral infarction classification; NIHSS = National Institutes of Health Stroke Scale.
